# Blind classification of e-scooter trips according to their relationship with public transport

**DOI:** 10.1007/s11116-023-10382-4

**Published:** 2023-03-16

**Authors:** Juan José Vinagre Díaz, Rubén Fernández Pozo, Ana Belén Rodríguez González, Mark Richard Wilby, Bani Anvari

**Affiliations:** 1 Department of Mathematics Applied to Information and Communication Technologies, Universidad Politécnica de Madrid, Avda. Complutense, 30, 28040 Madrid, Spain; 2 Centre for Transport Studies, Department of Civil, Environmental and Geomatic Engineering, University College London, WC1E 6BT London, UK

**Keywords:** Micromobility, Electric scooter, Rome, Public transit, Autonomous clustering

## Abstract

E-scooter services have multiplied worldwide as a form of urban transport. Their use has grown so quickly that policymakers and researchers still need to understand their interrelation with other transport modes. At present, e-scooter services are primarily seen as a first-and-last-mile solution for public transport. However, we demonstrate that $50\,\%$ of e-scooter trips are either substituting it or covering areas with little public transportation infrastructure. To this end, we have developed a novel data-driven methodology that autonomously classifies e-scooter trips according to their relation to public transit. Instead of predefined design criteria, the blind nature of our approach extracts the city’s intrinsic parameters from real data. We applied this methodology to Rome (Italy), and our findings reveal that e-scooters provide specific mobility solutions in areas with particular needs. Thus, we believe that the proposed methodology will contribute to the understanding of e-scooter services as part of shared urban mobility.

## Introduction

In recent years, the concept of micromobility, i.e. the type of shared mobility based on lightweight and unipersonal vehicles, has emerged as an important component of the transportation system in modern cities throughout the world (Clewlow 2019). Electric scooter (e-scooter) services have arisen as a novel and popular mode of travel, attracting new companies and investments to the rapidly growing shared mobility market (Voytenko Palgan et al. 2021).

The popularity of the e-scooter phenomenon and its exponential expansion across urban streets has motivated contradictory reactions (EIT Urban Mobility 2020). On the one hand, supporters highlight the advantages of having an easy to ride and enjoyable new form of urban mobility that provides sustainability benefits, such as reduced carbon emissions and energy costs (Hollingsworth et al. 2019). On the other hand, critics commonly object to concerns such as the increase of accidents (Badeau et al. 2019; Yang et al. 2020), parking issues on sidewalks and dedicated lanes (Fang et al. 2018), limited availability outside downtown areas (Ciociola et al. 2020; Masoud et al. 2019), and restrictions on the number of passengers and goods (Gössling 2020).

Some of these disadvantages result from the rapid emergence of e-scooters and the lack of the appropriate policy guidelines and regulations required to ensure their proper integration into the existing urban transportation system (Shaheen and Cohen 2019). To fulfill this need, we must extend our current knowledge about the way e-scooters are being used and their interaction with other modes of transport. Our work contributes to this objective by providing insights about the different roles e-scooters play in relation to public transport.

E-scooter trips can only be categorized into four possible classes, acting as (i) complementary, auxiliary on the (ii) first or (iii) last mile, or substitution (iv) to public transport. We could infer that an e-scooter has been used in connection with the public transport network if it was taken or left in the proximity of a station. The problem is defining *proximity* given that it depends on a set of complex factors including urban design, public transport network, or users’ behavior among others. Research in the field has opted for fixing a predefined maximum distance threshold to accept a connection between e-scooter trips and public transport. This methodology is arbitrary to some extent. To solve this issue, we have employed a *blind* spatial clustering technique to perform an autonomous classification of e-scooter trips. The term *blind* refers to the fact that we do not apply any *a priori* value for the maximum distance. Instead, we directly apply the clustering algorithm on real data to autonomously create clusters of trips with certain similarities. This way, the specific value of the maximum distance emerges from the data rather than being imposed. Consequently, our methodology may contribute to characterize the city’s behavior towards micromobility.

To this end, a data-driven approach is adopted to avoid potentially biased conclusions resulting from surveys or simulations. We chose Rome (Italy) as an optimal test scenario given that it has a broad public transport network, and, at the same time, shared mobility systems have been accepted by citizens. In addition, the majority of e-scooter studies have focused on cities in the USA, such as Austin (Bai et al. 2021; Caspi et al. 2020; Jiao and Bai 2020; Zuniga-Garcia and Machemehl 2020), Louisville (Hosseinzadeh et al. 2021; Noland 2019; Reck et al. 2021a), Chicago (Tuli et al. 2021), Indianapolis (Mathew et al. 2019), Washington (Hawa et al. 2021; McKenzie 2019; Merlin et al. 2021; Younes et al. 2020), and Atlanta (Espinoza et al. 2019), including several comparative analyses (Bai and Jiao 2020; Huo et al. 2021). Although cities outside the USA such as Zurich (Reck et al. 2021) or Singapore (Zhu et al. 2020) have been studied, little attention has been given to e-scooter use in other parts of the world. Our study in Rome contributes to fill up this gap.

Our study provides significant contributions to the scientific development in the field of shared mobility systems, including:

- An autonomous classification of e-scooter trips based on their interaction with the public transportation system. For this purpose, we used a modeling framework based on clustering techniques using the distances between e-scooters’ origins and destinations and public transportation stations.
- A study about micromobility in a European city, which complements the conclusions of previous research based in American cities.
- An analysis about how e-scooters fulfill the mobility needs of urban areas with specific requirements and characteristics, such as those with lower penetration of public transport.
- A new e-scooter database containing extended information about e-scooter trips in the city of Rome. We developed software to retrieve detailed information about e-scooter journeys, including trajectories with high temporal resolution and the state of the vehicle.

The remainder of the paper is structured as follows: Section "Related Work " presents related work about e-scooter trip characteristics and the relationship between micromobility and modes of public transit. Next, Section "Framework and methodology" describes the methodological framework we developed to study the relationship between e-scooters and public transport. Section "Empirical results: relationship between e-scooters and public transport" presents the empirical results we obtained by applying an e-scooter trips data set to the developed methodology. Section "Discussion" analyzes the spatial distribution of each type of trip and their statistical characteristics, comparing the results with related works. Finally, Section "Conclusions" provides the conclusions and highlights future research to emerge from this research.

## Related work

We first discuss existing research about factors influencing e-scooters’ use and the characteristics of their users. The relationship between micromobility and public transit will be described in the second subsection.

### E-scooters’ trip characteristics and users

Detailed knowledge about how citizens use e-scooters is key for transportation planners and policymakers. However, given the recent provision of this type of mobility services, most of the current research is based on surveys in American cities to estimate their demand (Clewlow 2019). Survey results generally report that commuting and leisure are equal purposes for e-scooter trips in Baltimore (Baltimore City Department of Transportation 2019), Portland (Portland Bureau of Transportation 2019), and San Francisco (San Francisco Municipal Transportation Agency 2019). In addition, questionnaires reveal that, on average, e-scooter users are young and college-educated men with incomes higher than the median of the area. Despite the global significance of these findings, they could be potentially biased due to their small sample size. Thus, mobility managers demand empirical studies based on real data to support their decision-making processes.

Research about shared e-scooter travel behavior has traditionally focused on modeling demand as the dependent variable, employing a set of explanatory variables from diverse multi-source data. These explanatory variables that can influence e-scooter usage can be categorized as trip-related (e.g., distance, time of day, destinations), external (e.g., built environment, land use, relationship with transit, weather) and internal (e.g., user socio-demographics, attitudes) (Reck et al. 2021; Tuli et al. 2021). For the first set of factors, the temporal characteristics of e-scooters’ demand were analyzed in Washington (McKenzie 2019) and Louisville (Noland 2019), confirming that they are mainly used for very short leisure journeys (usually less than a mile). From a temporal perspective, peaks during the afternoon are the most common (Mathew et al. 2019; Reck et al. 2021a) although some research has observed typical commuting behaviors (Caspi et al. 2020; McKenzie 2019). Regarding external factors, adverse weather conditions, such as precipitation or high wind speeds, have a negative impact on the demand for e-scooters (Noland 2019; Tuli et al. 2021), while warmer temperatures and better visibility are associated with high levels of usage (Younes et al. 2020). In addition, there are particular regions with greater demand, e.g., downtown areas and university campuses (Bai and Jiao 2020; Caspi et al. 2020; Hosseinzadeh et al. 2021; Jiao and Bai 2020; Mathew et al. 2019), business districts (Bai and Jiao 2020), and recreational or touristic neighborhoods (Merlin et al. 2021). These works show city-specific behaviors that are sometimes contradictory (Bai and Jiao 2020).

Beyond the general conclusions provided by this previous research, we are specifically interested in the interaction of e-scooter services and public transportation, as discussed in the next section.

### Relationship between shared micromobility and public transport

Micromobility plays an important role in connecting with the existing public transit system, acting as complementary (i.e., providing service to districts with little or no public transport), auxiliary (covering first and last-mile trips), or as a substitute (replacing public transport journeys) (Kong et al. 2020). Previous studies are inclined to treat micromobility as an auxiliary mode that facilitates the connection to public transport by providing a means to travel stretches beyond walking distance (Shaheen and Chan 2016; Smith and Schwieterman 2018; Lee et al. 2021). This role of micromobility has been observed during the evaluation of e-scooter pilot programs in Portland (Portland Bureau of Transportation 2019) and San Francisco (San Francisco Municipal Transportation Agency 2019) and bicycle sharing systems in cities worldwide, such as Beijing (Zhao and Li 2017), Washington (Ma et al. 2015), Chicago (Faghih-Imani and Eluru 2015), Vienna (Leth et al. 2017; Shaheen and Chan 2016), and Helsinki (Jäppinen et al. 2013).

However, the precise interrelation between micromobility and public transportation is far richer and more complex; complementary, auxiliary, and substitute roles are not mutually exclusive, and vary from city to city and even among modes of public transport. Currently, several investigations have observed micromobility as an auxiliary mode of subways and railways (Jin et al. 2018; Zhang et al. 2019), whilst operating as a substitute for public buses (Campbell and Brakewood 2017; Yang et al. 2018; Luo et al. 2021; Zuniga-Garcia and Machemehl 2020; Nikiforiadis et al. 2021; Kopplin et al. 2021; Laa and Leth 2020). In addition, modal substitution primarily occurs in densely populated urban cores (Martin and Shaheen 2014; Cao et al. 2021), while complementary and auxiliary effects were more frequent in districts with greater distances to public transport stations (Radzimski and Dzięcielski 2021; Shaheen and Chan 2016). On the other hand, other factors like the temporal components (e.g., weekday or weekend, time) and the type of user (frequent or occasional) are also key to identifying the particular role played by micromobility. Consequently, we often find a mix of micromobility roles within a single city (Yan et al. 2021), and further research is required to precisely characterize the interactions between micromobility and public transportation systems (Espinoza et al. 2019).

Our work focuses on solving the issue of classifying the interrelation between e-scooter services and public transportation, developing an algorithm that autonomously separates e-scooter trips into complementary, auxiliary, or substitute roles without *a priori* information. To determine the role played by a micromobility trip, the majority of previous research relies on the distance between the origin or destination of the route and the corresponding closest station in the public transportation network. Such an approach requires fixing a set of predefined thresholds to separate the measured distances. However, there is no consensus about this matter. Instead of being treated as a *design parameter* of the classification algorithm, this predetermined distance may rather be considered as a *feature* of the specific city and transport system, and thus capable of representing the particular use of micromobility.

## Framework and methodology

### Public transport and e-scooter service in Rome

**Fig. 1:**
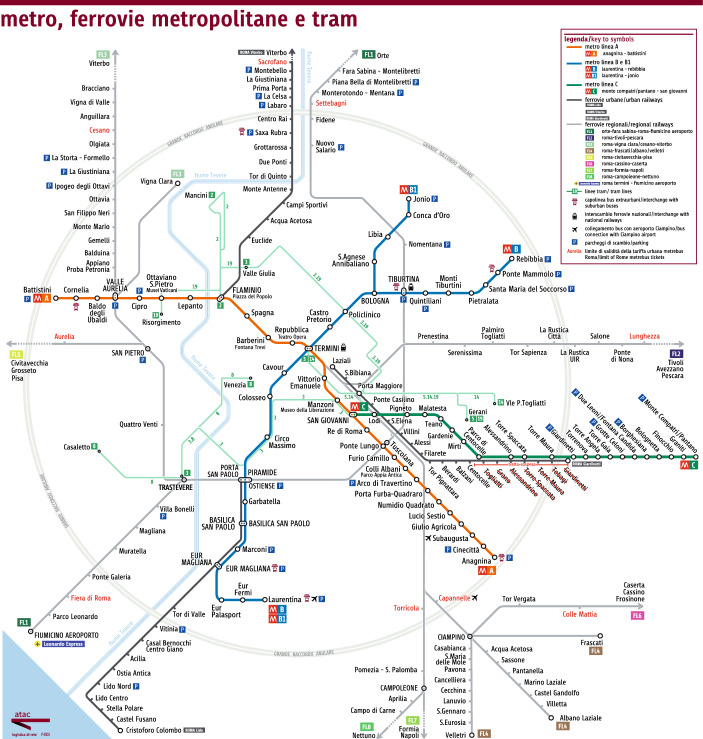
Subway, train and tram map of Rome. URL https://www.atac.roma.it/docs/default-source/mappe-tpl/mappa-metro-e-ferrovie-metropolitane.pdf?sfvrsn=e1e83890_16

The capital city of Rome is located in central Italy and is the largest city in the country, with a population of approximately 2.8 million residents according to the 2021 census. The city is a popular tourist destination, attracting millions of tourists every year. Rome has an extensive internal transportation system, although mobility is primarily based on private vehicles, which creates frequent congestions throughout the road network. Rome’s public transit system consists of subway, rail, bus, and tram services, connecting every location in the city (Cipriani et al. 2019). In particular, Rome’s subway includes three underground lines and 73 stations; its overground rail transport comprises the tram network, and seven suburban and urban train lines that connect the surrounding areas to the city; the bus network has broad coverage (338 lines), but with low to medium frequency of service. The *Mobility Agency of Rome*^1^ is the municipal public transport agency in charge of providing information and services to the user. Public transit routes, stops, and real-time schedule data are publicly available in the open data section of the agency’s website and provided using the General Transit Feed Specification (GTFS) format. Through this open data portal, we downloaded and processed the data necessary to obtain geographic locations (longitudes and latitudes) of train and subway stops in the public transport network (Fig. 1)

The e-scooter sharing market in Italy is still in a pre-competitive stage, meaning that there are no nationwide regulations (Carrese et al. 2021). Between June 2019 and February 2020, Italy adopted and reformed its micromobility regulation, where e-scooters are considered as bikes in terms of circulation, with a maximum allowable speed of 20 km/h (reduced to 6 km/h in pedestrian areas). These laws were recently updated (November 2021) to introduce new rules: users are no longer allowed to travel or park on sidewalks in an attempt to clamp down on the "*wild way*" the scooters are being parked^2^. The first e-scooter sharing services started operating in major Italian cities in December 2019, but the service in Rome was launched in May 2020, coinciding with the start of the COVID-19 emergency (Carrese et al. 2021). Rome followed the adoption approaches of other Italian cities like Milan and Turin, setting up a maximum number of e-scooter sharing operators to guarantee competition between them. Eight licensed sharing companies are currently active in Rome: Dott, Lime, Bird, Wind, Link, Voi, Keri, and Helbiz, whose data will be used in this work. Helbiz provides good coverage around the city, including the restricted access zone (RAZ) in Rome’s downtown, and provides an additional service to the southwest area compared to other operators.

### Methodology: autonomous classification of trips

The purpose of this work is to construct an unsupervised method to extract whether e-scooters interact with the existing public transit network as a complementary (filling the gaps of public transport), auxiliary (connecting to the public transport network at the origin or the destination), or substitute (replacing public transport) mode. To this end, we will study the distance between the origin and destination of e-scooter trips and the closest train or subway station, following the usual approach outlined in the literature we described in Section "Related Work ". In the absence of precise information about the actual role each micromobility trip played, this minimum distance is the best approach researchers can take in order to infer the underlying interaction with public transport. Even considering potential errors in the absolute number of connections, they still be valid in relative terms, thus acting as metrics to compare different scenarios and monitor the evolution of the roles micromobility plays in association with public transport. However, instead of using a predefined and arbitrary threshold to determine this measure of closeness as other works in the field do, we developed a methodology based on a clustering approach that allows the *autonomous* or *blind* classification of trips.

Clustering is a complex task that entails a set of challenges (Jain and Dubes 1988), thus being one of the major issues in machine learning. It has many applications arising from different disciplines, including Smart Mobility (Vinagre Díaz et al. 2020). Clustering involves partitioning a given data set into subsets based on the closeness or similarity among the data (Peng and Xia 2005). Typically, the similarities among entities in a data set are measured by a specific proximity function, which can be calculated in many ways, each of which results in a different clustering algorithm. Most clustering algorithms belong to one of two classes: hierarchical clustering or partitioning. The hierarchical approach produces a nested series of partitions, consisting of clusters either disjointed or included one into the other. These algorithms begin by considering every entity as a cluster, and then proceed by successively merging clusters using an objective function until a stopping criterion is reached. In contrast, partitioning methods assume a given number of clusters to be found and then look for the optimal partition based on an error function. The most commonly used approach among these methods is the well-known K-means (Jain and Dubes 1988), which is capable of providing the same level of performance as other hierarchical algorithms and DBSCAN (Ester et al. 1996) at a much lower computational cost.

Euclidean distances from each entity to its assigned cluster center are the most broadly used criterion in clustering (Peng and Xia 2005). However, more specific metrics could better fit certain applications. For example, if the variable of interest is a geographical distance (as in our case), the Manhattan distance is a more appropriate candidate. The Manhattan distance is a metric in which the distance between two points is calculated as the sum of the absolute differences of their Cartesian coordinates. In other words, it is a measure of the distance in a grid layout, where diagonal “movements” are not allowed. Its name comes from the grid layout of Manhattan’s streets.

Formally, consider a general data set ${\mathcal {S}}$ with *n* samples in a *d*-dimensional space, denoted by:

$$\begin{aligned} {\mathcal {S}} = \left\{ s_1, s_2,\ldots , s_n\right\} ,\; s_i \in {\mathbb {R}}^d. \end{aligned}$$

The task of K-means is to assign each of the *n* samples in ${\mathcal {S}}$ to *k* disjoint clusters $S_j$, with centroids $c_j, \; j=1,\ldots , k$ and ${\mathcal {S}} = \bigcup \limits _{j=1}^{k} S_j$, such that a clustering criterion is optimized. Therefore, K-means solves an optimization problem, selecting the specific partition ${\mathcal {S}}^{p} = \left\{ S^{p}_{1}, S^{p}_{2}, \ldots , S^{p}_{k}\right\}$ of the complete data set ${\mathcal {S}}$, which minimizes an error function $e({\mathcal {S}}^{p})$.

In our case, samples are 2-dimensional vectors $\textbf{v}=(v_1,v_2) \in {\mathbb {R}}^2$, whose components take the value of the distance from the origin ($v_1$) and the destination ($v_2$) of the e-scooter trip to the closest subway or train station. In addition, we define an error function, based on the Manhattan distance, calculated for each partition ${\mathcal {S}}^{p}$ as:

$$\begin{aligned} e({\mathcal {S}}^{p}) = \sum _{j=1}^{k} \sum _{i=1}^{|S^{p}_{j}|} ||s_{ij} - c_j|| \, , \end{aligned}$$

where $|S^{p}_{j}|$ is the number of samples in subset $S^{p}_{j} \in {\mathcal {S}}^{p}$, with centroid $c_j$, $s_ {ij}$ is the *i*-th sample in $S^{p}_{j}$, and $||s_{ij} - c_j||$ denotes the Manhattan distance between each sample in the subset and its centroid. Note that the Manhattan distance calculates the distance between two samples by aggregating the pairwise absolute difference between each variable, while Euclidean distance aggregates the squared differences.

Therefore, our methodology can be described as the following bi-level programming problem:

$$\begin{aligned} \min _{c_i,...,c_k} \sum _{i=1}^{n} \min {\left\{ ||s_{ij} - c_1||, ... ,||s_{ij} - c_k||\right\} }, \end{aligned}$$

that we solve using a K-means algorithm:

- *INPUT*: data set ${\mathcal {S}}$ with *n* samples; *k* number of clusters.
- *OUTPUT*: partition $${\mathcal {S}}^{p} = \left\{ S^{p}_{1}, S^{p}_{2}, \ldots , S^{p}_{k}\right\}$$.
  1. Choose *k* cluster centroids $c_j (j=1,...,k)$ randomly generated in a domain containing all *n* samples.
  2. Assign each sample to the closest cluster centroid, creating partition ${\mathcal {S}}^{p}$.
  3. Recompute the cluster centroids using the current cluster memberships.
  4. Calculate the error function $e({\mathcal {S}}^{p})$.
  5. If a convergence criterion is met, stop; otherwise go to step 2.
  6. Return output $${\mathcal {S}}^{p}$$.

This methodology was implemented in the Matlab programming language. The obtained results will be presented and discussed in Section ""4.

## Empirical results: relationship between e-scooters and public transport

### E-scooter trips data set

Using the described methodology, we assigned each e-scooter trip to a specific class depending on how it relates to the public transport system. Given the data-driven nature of our approach, we built a new e-scooter database for Rome (Italy) and developed a customized Python code that interacts with the Helbiz API^3^. Every 10 seconds, this software collects information about each scooter: its vehicle identifier, the longitude and latitude coordinates of its position, and four boolean flags. The latter allow an inference about its state: in use, parked, or out of service. The process ran during February 2021 (28 days) and collected around 624 million records. In total, $25\,186$ e-scooter trips were found.

### Data filtering

To guarantee data quality, we first filter out records that may result from the inherent limitations of GPS technology or other sources of error in the acquisition process, which could eventually compromise the feasibility of a precise spatio-temporal analysis. Related literature in the field focuses on eliminating outliers by applying filters to traveled distance, duration, and speed. We chose the filtering criteria in (Zou et al. 2020), thus accepting trips with a traveled distance between 100 m and 20 kilometers, a time duration between 30 seconds and 125 minutes, and an average speed below 25 km/h, which is just above the maximum speed allowed.

This cleaning process resulted in a robust data set of $23\,690$ valid trips. Less than $6\,\%$ of the original user trips ($1\,496$) were removed, the majority of which were due to not exceeding the lower distance boundary. During the period of study, $2\,559$ different Helbiz e-scooters were in operation, which indicates that this operator has a significant fleet size.

**Fig. 2:**
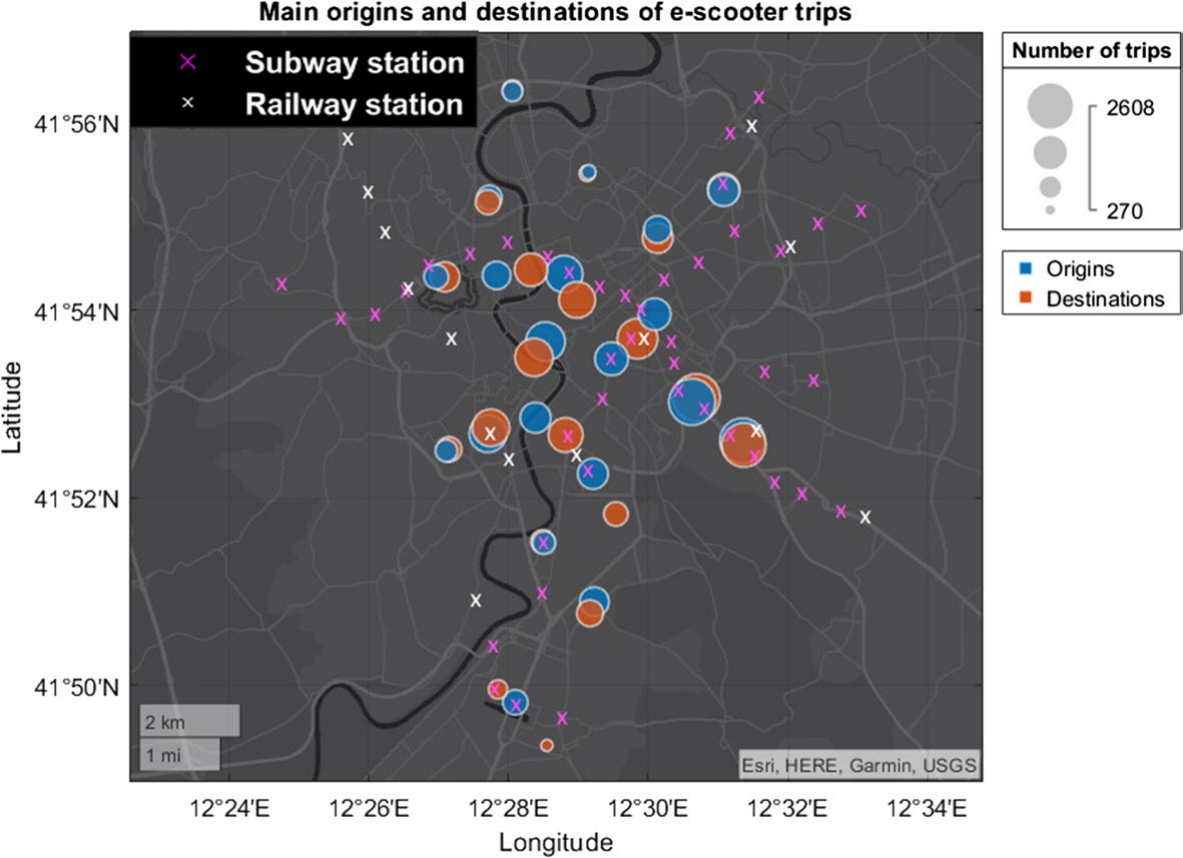
Main origins and destinations of e-scooter trips in Rome

The origins (blue) and destinations (red) of the resulting valid trips are depicted in Fig. 2, where the size of the circles indicates the volume of trips starting or ending at that location.

### Empirical implementation and results

As described in Sect. "Methodology: autonomous classification of trips", our objective is to classify e-scooter trips as a complementary, auxiliary, or a substitute mode of transport in relation to the public transport system. In this regard, our analysis focuses on subway and railway systems for several reasons. First, previous literature confirms that travelers often use shared micromobility options to connect with primarily rail services rather than buses (Moinse et al. 2022; Martens 2004; Martin and Shaheen 2014), except for suburban areas with a low bus coverage (Luo et al. 2021), which fall outside of the scope of this study. Second, in those areas where e-scooters and buses coexist, the former often substitutes the latter given that they provide a more flexible mode of transport to cover similar typical trip distances (Campbell and Brakewood 2017). Third, the bus network in Rome is geographically extensive, with an extremely high number of stops throughout the city, which would significantly increase the probability of finding a bus stop close to the origin or destination of every e-scooter trip; subsequently, all the trips would erroneously be classified as auxiliary. Therefore, our method studies the distance between the origin and destination of e-scooter trips and the closest train or subway station, following the usual approach in the literature. However, instead of using a pre-determined and arbitrary threshold to determine this measure of closeness, we will use the clustering methodology described in Sect. "Methodology: autonomous classification of trips" to empirically discover the boundaries between classes, thus achieving an *autonomous* classification of trips.

**Fig. 3:**
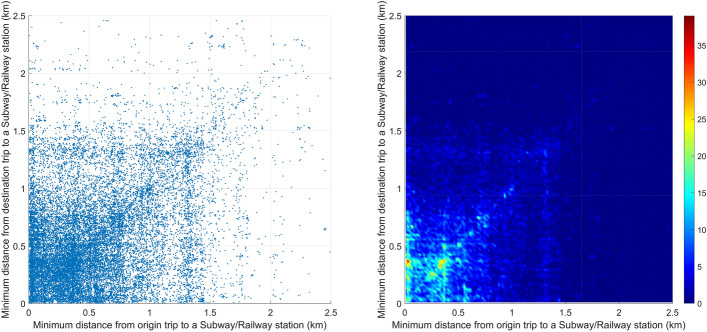
Scatter plot (left) and density plot (right) of minimum distances from the origin and destination of e-scooter trips to a railway or subway station

**Fig. 4:**
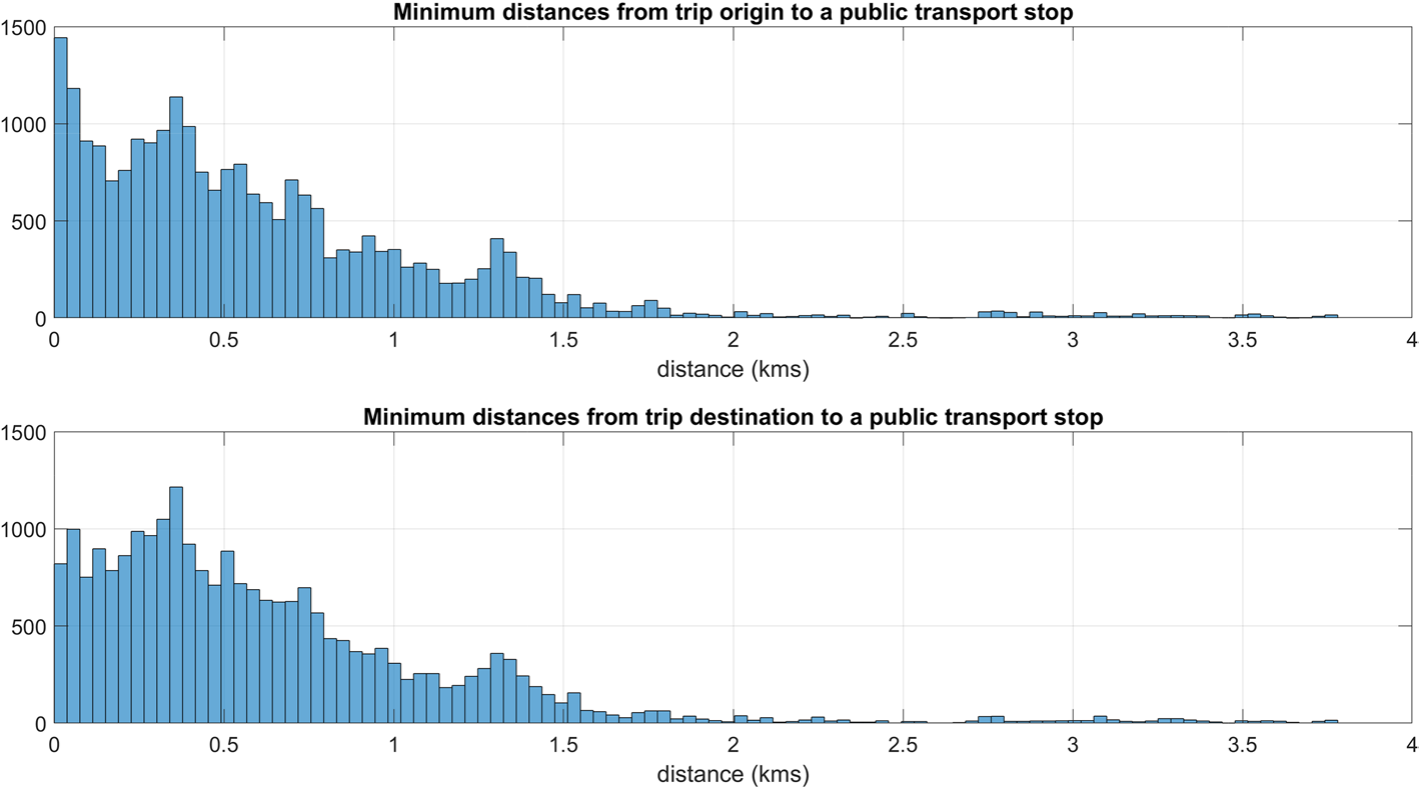
Distributions of minimum distances from trip origin and destination to subway and railway stations

This empirical approach consists of two phases. In the first phase, for each e-scooter trip, we calculate the minimum distance from its origin and its destination to every geographical location of subway and railway stations in the public network. Figure 3 shows the scatter plot (left) and the density plot (right) of the minimum distances from the origin (*x*-axis) and the destination (*y*-axis) of e-scooter trips to the closest railway or subway station; therefore, each point represents one of the $23\,690$ e-scooter trips in the data set ${\mathcal {S}}$. In addition, we can observe in Fig. 4 the statistical distributions of these distances from origin (top) and destination (bottom) to the closest public transport station. Recent approaches in the field such as (Kong et al. 2020), (Lv et al. 2021), or (Yan et al. 2021), apply a fixed threshold to the minimum distance, which varies from 400 to 600 m and aims at reflecting the comfortable walking distance for users. This range of distances includes the highest frequencies in Fig. 4, which suggests the actual existence of an underlying connection between e-scooter trips and public transport that depends on this minimum distance. However, the graph does not show a definite value for this minimum distance, which will in addition have a significant effect on the results of the study as we will discuss in Sect. "Discussion".

In the second phase, we apply the blind clustering algorithm described in Sect. "Methodology: autonomous classification of trips" to this 2-D data set to classify e-scooter trips depending on their relationship with the existing public transit network. In order to reflect the roles of micromobility related to public transport, researchers in the field choose a predefined number of clusters depending on their specific study. Some works use 2 classes that correspond to two specific behaviors: competition (class A), or supplementary (class B) (Leth et al. 2017); or whether shared mobility integrates (class A) or not (class B) with public transport (Gössling 2020). Other authors use 3 classes to reflect competition (class A), supplementary (class B), and auxiliary (class C) purposes, with no detail about auxiliary trips for the first and last mile (Kong et al. 2020). In our case, we select 4 classes to represent all four possible behaviors: (1) complementary, auxiliary (2) at origin or (3) at destination; and (4) substitute.

**Fig. 5:**
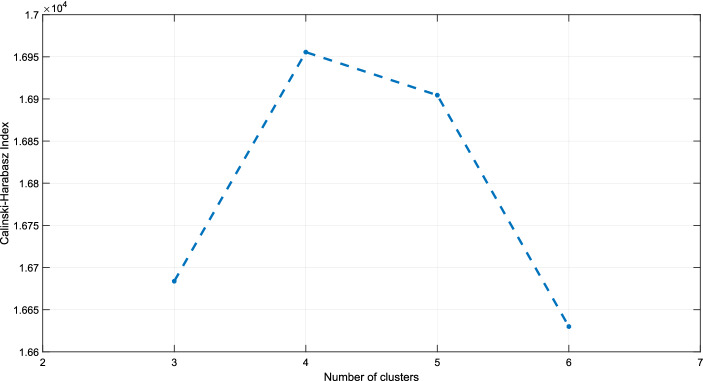
Calinski-Harabasz Index for evaluating cohesion and separation of clustering results depending on the number of classes

Consequently, we fix the number of clusters $k = 4$ of our method. This decision was validated using the Caliński-Harabasz (CH) Index (Caliński and Harabasz 1974), specifically tailored for situations in which ground truth labels are unknown as in our case study. The CH Index measures the cohesion and separation of clusters. Clustering methods reaching high values of the CH Index are proved to be capable of creating dense and well separated clusters. The results of this test are shown in Fig. 5, where we confirm that the best candidate is $k=4$.

**Fig. 6:**
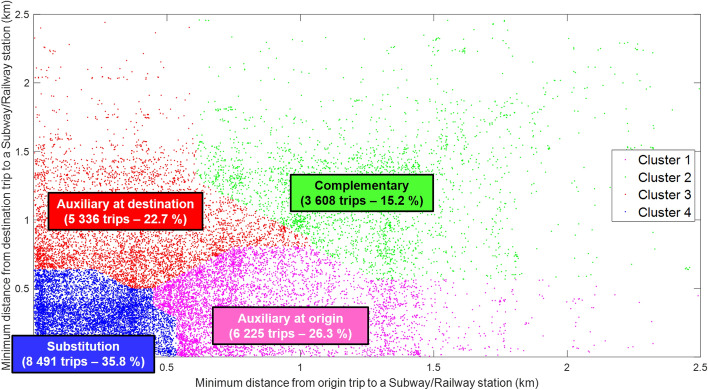
Result of the autonomous classification of e-scooter trips in Rome

Figure 6 shows the result of our autonomous classification of e-scooter trips applied to the data set collected in Rome. This method is capable of autonomously partitioning the original data set into sensible and explicable clusters. Thus, complementary trips (green cluster) are characterized by having both an origin and destination far away from a railway or subway station; in this case, the e-scooters are used to complement public transport, providing mobility services to areas with low coverage of the public network. Auxiliary trips are characterized by serving as a connection to public transport either at the origin (magenta cluster) or the destination (red cluster). Finally, substitute trips (blue cluster) are those where the traveler preferred to use an e-scooter instead of a viable journey on public transport, thus showing both origin and destination close to a railway or subway station.

In the next section we discuss these results and compare them to other related approaches.

## Discussion

The classification of e-scooter trips serves as the basis for a deeper analysis on the use of this form of transport as part of the overall mobility in the city. In general, the results show that the e-scooter services in Rome are simultaneously competing with and supporting public transport given that the corresponding substitute and auxiliary clusters account for almost $90\,\%$ of the total trips. Despite e-scooter services providing a popular solution to accessing last-mile public transport, this indicates that more than $50\,\%$ of the trips are not related to this auxiliary purpose. This finding reveals the importance of e-scooter services as substitutions for and complementary to public transport, thus calling into question the conclusion of previous works that restrict their use to first and last-mile support.

### Patterns on the distance measurements

First, it is necessary to examine the distance measurements that relate e-scooter trips and public transport stations. The density plot in Fig. 3 shows the presence of *vertical* and *horizontal* sets of trips. Trips falling in one of these sets share a common distance to a public transport station, either from its origin (vertical) or destination (horizontal). This suggests that each e-scooter trip started or ended at a relevant point of interest that generates a high demand, located at the corresponding distance from its closest public station. We checked this hypothesis by analyzing these specific sets of trips. As an illustrative example, let us concentrate on the horizontal line approximately at 1.3 kilometers on the *y*-axis. The majority of the destinations of the e-scooter trips in that set are close to a park, Tenuta di Tormarancia (marked as A in Fig. 8), which is 1.3 kilometers away from its closest subway station, Marconi (marked as B in Fig. 8). These trips started almost anywhere in the city, but they all shared a common destination, the park, which is at that specific distance from its closest public transport station.

Furthermore, the distribution presents a diagonal symmetry. This shows that users frequently use e-scooters for round trips, where the destination of the outbound journey coincides with the origin of the return journey, thus generating two symmetric trips. This is typical commuting behavior.

Finally, a diagonal for distances below 1 km is also observed. This feature suggests short and circular e-scooter trips that started and ended at roughly the same location. Given the availability of trajectory information in the data set we constructed, we confirmed this hypothesis by analyzing their detailed path. Short and circular trips are also typical e-scooter user behavior for leisure purposes.

### Spatial distribution of trips by their class

**Fig. 7:**
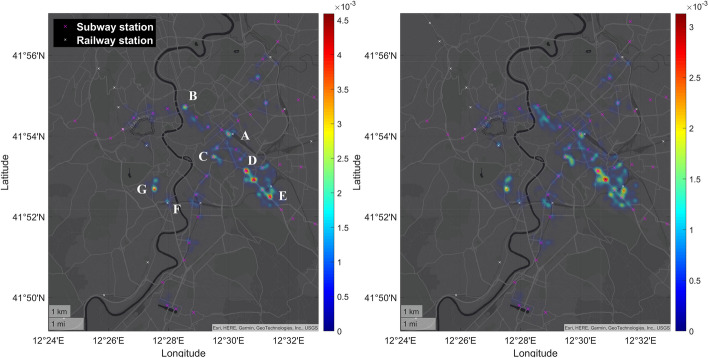
Spatial distribution of *substitution trips*: origins (left) and destinations (right). A: Termini; B: Flaminio; C: Colosseo; D: San Giovanni; E: Furio Camillo; F: Roma Trastevere; G: Quattro Venti

**Fig. 8:**
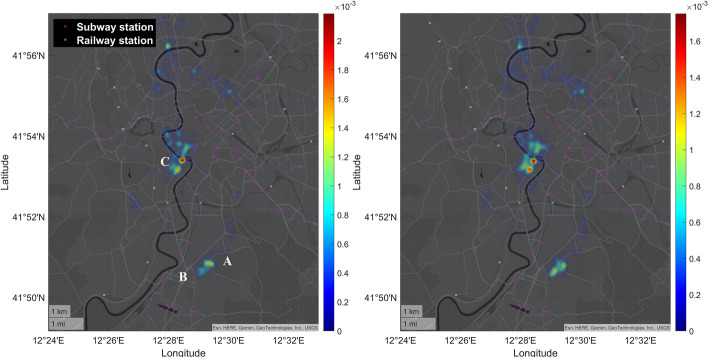
Spatial distribution of *complementary trips*: origins (left) and destinations (right). A: Via Attilio Ambrosini; B: Via Crostoforo Colombo; C: Trastevere

**Fig. 9:**
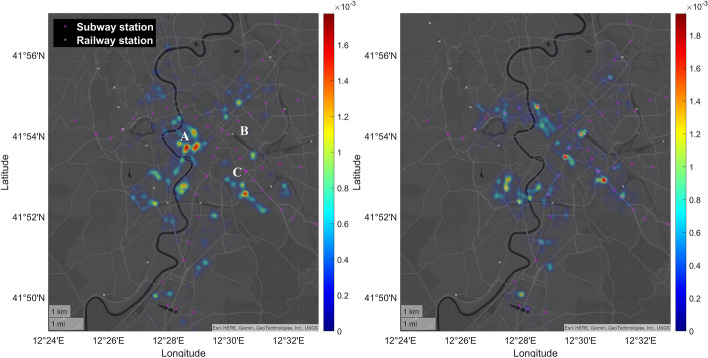
Spatial distribution of *auxiliary trips at origin*: origins (left) and destinations (right). A: Historical downtown; B: Termini; C: San Giovanni

**Fig. 10:**
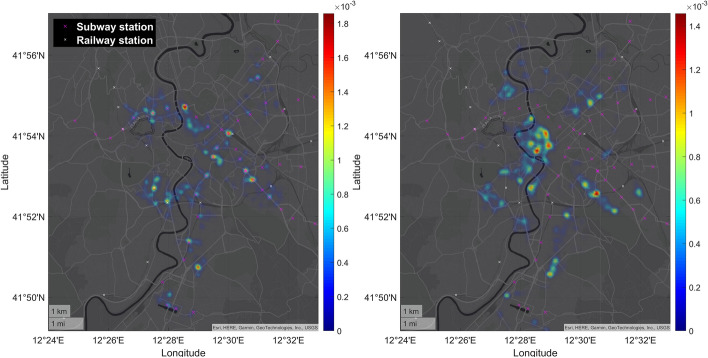
Spatial distribution of *auxiliary trips at destination*: origins (left) and destinations (right)

The classification of e-scooter trips allows us to perform a spatial analysis of this type of micromobility to understand which type of service e-scooters provide to different areas of the city. The set of figures comprising Figs. 7– 10 show the spatial distribution of the origins (left) and destinations (right) of e-scooter trips operating as a substitute, complementary, or auxiliary at origin or destination, respectively. A general observation of these figures indicates that there are specific uses of e-scooters in specific areas of the city, which provides a more complete picture for both urban and transport planners and micromobility operators.

E-scooters are mainly used as a substitute transport mode in four sections of the public transport network (all stations are marked on Fig. 7): subway line A connecting Termini (place A) with Flaminio (place B), subway line B connecting Termini with Colosseo (place C), subway line A connecting San Giovanni (place D) with Furio Camillo (place E), and railway connecting Roma Trastevere (place F) with Quattro Venti (place G). The first three sections include some of the most frequently used subway stations in Rome; consequently, our results show that e-scooters are accepted by users as an optimal transport mode to avoid crowded environments. There is only a railway link between Roma Trastevere and Quattro Venti with a low frequency of trains and an average waiting time of 15 minutes; users prefer to take the e-scooter to cover the distance in less than 4 minutes rather than waiting for a train to arrive.

On the other hand, complementary e-scooter trips are distributed throughout the areas in the city with lower access to public transport. As observed in Fig. 8 the shaded areas cover urban zones that are distant from the railway and subway networks. This is significant in the surroundings of Via Attilio Ambrosini (place A) and the Trastevere (place C). The former is a residential area, full of small shops, banks, and local trade, where neighborhoods benefit from the flexibility of e-scooters for their mobility. The latter is a lively area of Rome, with restaurants and bars that require good transport connections to the rest of the city.

Finally, the spatial distribution of auxiliary e-scooter trips presents symmetrical behavior observed in the left graph of Fig. 9 and the right graph in Fig. 10. This means that e-scooter services in these areas cover the user needs for the first and last mile of public transport journeys. In addition, the symmetry we observe fits with commuter trips that merge public transport and micromobility. This type of behavior is mostly seen in the historical downtown (place A) and some of the main transport hubs in Rome such as Termini (place B) or San Giovanni (place C).

### Statistical analysis of each class of trip

Table 1 shows the statistical characterization for traveled distance, duration, and speed of trips belonging to each class regarding their relationship with public transport. For each magnitude, we indicate mean, standard deviation minimum and maximum values. Overall, mean values for each class are similar across all groups, with no significant differences. This prevents these features from being used as direct classifiers of trips. These results show that complementary trips are the slowest, while substitution trips are the shortest in duration and traveled distance. Both findings are plausible: complementary trips provide a mobility service in areas with a low penetration of public transport and also include leisure journeys that are expected to have a lower speed. Substitute trips mainly occur for short distances, where e-scooters are able to compensate for waiting times at public transport stations.

**Table 1:** Statistical characterization for basic features in every e-scooter trip class

	Distance (m)
All trips	$2\,053.0 \pm 1\,633.0 \quad \quad [100.1 \quad 18\,268.1]$
Auxiliary at origin	$2\,235.9 \pm 1\,663.9 \quad \quad [101.6 \quad 17\,694.7]$
Auxiliary at destination	$2\,336.0 \pm 1\,687.8 \quad \quad [105.1 \quad 18\,268.1]$
Complementary	$1\,964.9 \pm 1\,620.1 \quad \quad [100.1 \quad 16\,714.3]$
Substitute	${ {1\,777.6}} \pm 1\,530.1 \quad \quad [100.6 \quad 13\,999.2]$
	Duration (s)
All trips	$688.8 \pm 719.8 \quad \quad [30 \quad 7\,491]$
Auxiliary at origin	$730.0 \pm 727.6 \quad \quad [30 \quad 7\,380]$
Auxiliary at destination	$747.1 \pm 695.4 \quad \quad [31 \quad 7\,231]$
Complementary	$705.6 \pm 765.0 \quad \quad [30 \quad 7\,431]$
Substitute	${ {614.6}} \pm 703.1 \quad \quad [30 \quad 7\,491]$
	Speed (km/h)
All trips	$12.11 \pm 4.21 \quad \quad [0.05 \quad 23.18]$
Auxiliary at origin	$12.39 \pm 4.18 \quad \quad [0.08 \quad 23.18]$
Auxiliary at destination	$12.58 \pm 4.05 \quad \quad [0.37 \quad 22.69]$
Complementary	${{11.49}} \pm 4.38 \quad \quad [0.05 \quad 22.66]$
Substitute	$11.86 \pm 4.19 \quad \quad [0.06 \quad 22.86]$

### Comparison with related approaches

In this paper we have developed a new approach for analyzing the interactions between e-scooters and existing public transit using a blind clustering method. Most of the previous work about this issue examined the problem using predefined design criteria. For these works, the key factor is whether the e-scooter trip starts or ends inside or outside a certain coverage area surrounding the public transport station. They delimit this area using a predefined radius that represents the walking distance users would accept to get to the public transport. Nevertheless, there is no consensus among the scientific community about the specific value to assign to this radius: 400 m in (Kong et al. 2020), 600 m in (Lv et al. 2021), a quarter mile in (Yan et al. 2021), etc. There is no robust basis to select one value or another as it depends on people’s perception or preferences. In addition, a particular distance value that could fit the characteristics of a city could, however, be invalid for another. The major problem in this regard is that the direct classification of e-scooter trips depends on the value of this radius, and, subsequently, the conclusions that may be extracted from the corresponding results. In order to overcome this issue, we have developed a methodology that does not need to define any *a priori* parameter to analyze the relationship between e-scooter and public transit, and which classifies trips autonomously, deriving any required information from the original data.

To compare our results with other methodologies, we developed and applied an analytical approach adapted from the work of (Kong et al. 2020) to our data set, which infers the substituting or complementary relationship between bike-sharing systems and public transit based on distance thresholds. We define the transit coverage using some predefined values of the radius, from $R = 100$ m to $R = 500$ m, with the aim of evaluating the influence of this parameter. The distribution of trips among the three different categories (substitute, complementary, and auxiliary) is compared among each approach and illustrated in Table 2.

If 500 m is used as the radius, the number of trips belonging to each class is similar to our clustering results. This indicates our methodology is capable of autonomously determining this value, intrinsic to each city, without imposing any artificially predefined threshold (Fig. 6). However, the percentage of trips in each category resulting from the analytical method using, for example an *a priori* radius $R = 100$ m, are completely different. The majority of trips are classified as complementary because such a short radius makes it difficult to find trips that start or end near the public transport stations. This comparison highlights the risk of using an inappropriate radius to classify micromobility trips in regards to their interaction with public transport.

**Table 2:** Trip classification percentage comparing our clustering method with other analytical method based on different predefined parameters

Method	Substitution trips	Complementary trips	Auxiliary trips
Blind clustering	$35.84\,\%$	$15.23\,\%$	$48.93\,\%$
Analytical${\text{R}} = 500$ m	$30.06\,\%$	$26.59\,\%$	$39.35\,\%$
Analytical${\text{R}} = 300$ m	$11.73\,\%$	$49.84\,\%$	$38.43\,\%$
Analytical${\text{R}} = 200$ m	$4.75\,\%$	$63.32\,\%$	$31.92\,\%$
Analytical${\text{R}} = 100$ m	$2.17\,\%$	$77.47\,\%$	$20.36\,\%$

## Conclusions

E-scooter services have recently attracted attention due to the rapid increase of this novel mode of mobility. However, transportation planners and policymakers need to know the exact impact of e-scooter services on their transport systems in order to efficiently integrate them into urban mobility. The former require precise information in order to optimize the service from a temporal (schedules) and spatial (design of network of stations and routes) perspective (Oeschger et al. 2020). The latter must adapt the current policies and create new regulation in several areas such as fares (Lee et al. 2021), safety, traffic, and urban design (Bozzi and Aguilera 2021). Our study contributes to this topic with a data-driven methodology that can generate knowledge about the specific role played by e-scooters in association with public transport.

To this end, we collated a new e-scooter database in Rome, with some relevant novelties with respect to other data sets employed in previous research. We designed a novel modeling framework based on clustering techniques and Manhattan distances to autonomously classify e-scooter trips according to their relation to public transport. This blind methodology avoids the need for any predefined design criteria or *a priori* artificial selection of parameters as used in other research. In addition, its autonomous nature allows it to extract the city’s intrinsic behavior in regards to the distance users accept walking to access public transport.

Our analysis reveals that the majority of the e-scooter trips in Rome are connected with subway or railway stations, which suggests the existence of both competing and supporting effects in Rome. We have also compared the spatial distributions of different trip types showing that e-scooters adopt different roles in different areas of the city. We believe that this novel methodology and the conclusions of this research have significant implications for transportation researchers, policymakers, and transit agencies regarding the design and management of e-scooter systems and their interaction with public transit.

These promising results offer several opportunities to further extend this strand of work. We plan to extend this study to other operators and international cities to perform comparative analyses between them. Second, we will also expand the temporal extension of the original data set to look for seasonal effects. Third, given that the collected data contains valuable information about e-scooter trajectories, we plan to evolve the traditional analyses based on origin and destination to actual paths to identify e-scooter routes throughout the city. Finally, we also aim to study the utilization of the e-scooters fleet to quantify the viability of this service from an operating perspective (González et al. 2021) .

## Footnotes

1 https://romamobilita.it/en

2 https://www.wantedinrome.com/news/italy-updates-highway-code-with-new-rules-of-the-road.html

3 https://api.helbiz.com
